# Evaluating quality indicators in colorectal cancer screening via fecal immunochemical tests: a five-year study from a developing country

**DOI:** 10.1186/s12876-025-04527-1

**Published:** 2025-12-30

**Authors:** Elham Tabesh, Farimah Rahimi, Maryam Soheilipour, Neda Tavakoli-Moghadam, Zahra Ravankhah, Seyedeh-Zeynab Hosseinian, Peyman Adibi

**Affiliations:** 1 Isfahan Gastroenterology and Hepatology Research Center, Isfahan University of Medical Sciences, Isfahan, Iran; 2 Health Management and Economics Research Center, Isfahan University of Medical Sciences, Isfahan, Iran; 3 Department of Community and Family Medicine, School of Medicine, Isfahan University of Medical Sciences, Isfahan, Iran; 4 Cancer Registry of Health Deputy, Isfahan University of Medical Sciences, Isfahan, Iran

**Keywords:** Adenoma detection rate, Adenomas per colonoscopy, Colorectal cancer screening, Bowel preparation, Colonoscopy

## Abstract

**Background:**

Assessing quality indicators in colorectal cancer (CRC) screening via the fecal immunochemical test (FIT) is crucial. However, data on developing countries with lower CRC rates, lifestyle transitions, newly established screening programs, and no consensus on optimal targets are scarce.

**Methods:**

This analysis evaluated 2,209 average-risk individuals aged 50–70 years in Iran between 2017 and 2022. The polyp detection rate (PDR), adenoma detection rate (ADR), sessile serrated lesion detection rate (SDR), and adenomas per colonoscopy (APC) were calculated. Patient relationships with the center, colonoscopist, bowel preparation, and comorbidities were assessed. Additionally, potential risk factors for bowel preparation quality were examined.

**Results:**

The analysis revealed a PDR of 34.5%, an ADR of 25.3%, an advanced ADR of 10%, a proximal ADR of 13.0%, an SDR of 1%, and a CRC rate of 2.7%. After adjustment, adenoma risk was greater in men (OR 1.84; 95% CI 1.49–2.69; *p* < 0.01) and those aged 60–69 (OR 1.28; 95% CI 1.04–1.58; *p* = 0.02), with similar trends for other detection rates. Polyps but not adenomas were more common in public centers (OR 1.28; 95% CI 1.04–1.57; *p* = 0.02) and among the academic group (OR 1.34; 95% CI 1.07–1.68; *p* = 0.01). PDR and APC were higher in colonoscopies with cecal intubation (*p* < 0.05). Bowel preparation quality was higher in private centers, with academic physicians, and among patients with a BMI < 30 (*p* < 0.05).

**Conclusion:**

The results provide early evidence of proposed ADR targets in developing countries. Given that the ADR depends on background epidemiology, implementing a dynamic, standard living point system based on extensive data is crucial. We recommend updating physicians on screening guidelines, prioritizing men, older adults, diabetic patients, and obese individuals.

**Supplementary Information:**

The online version contains supplementary material available at 10.1186/s12876-025-04527-1.

## Introduction

Colorectal cancer (CRC) is currently the third most frequently diagnosed cancer globally [1, 2]. The incidence of CRC is on the rise worldwide, particularly in developing countries such as Iran, where a Western lifestyle is becoming more common [2, 3]. Approximately 70% of sporadic CRCs develop from adenomatous polyps, and others arise from sessile serrated lesions (SSLs) [4], making screening crucial for identifying and removing these precursor lesions to reduce CRC incidence and mortality [5, 6]. While direct screening colonoscopy is the preferred method, noninvasive screening modalities such as annual fecal immunochemical tests (FITs), followed by colonoscopy, have become a popular alternative due to their high sensitivity, specificity, and low cost [7].

Given that the effectiveness of colonoscopy can be influenced by various factors, quality indicators have received significant attention [8]. Various intraprocedure quality indicators, such as the polyp detection rate (PDR), adenoma detection rate (ADR), advanced adenoma detection rate (AADR), serrated adenoma detection rate (SDR), and adenoma per colonoscopy (APC), have been proposed [8, 9]. The term adenoma detection rate is used instead of adenoma prevalence rate because the number of adenomas reported reflects those detected rather than the total number present. Accordingly, most references prefer this term, which is widely adopted in the literature.

Adequate bowel preparation is also crucial for the quality of colonoscopy [8]. Among these factors, the ADR is well validated as a predictor of postcolonoscopy CRC, is easily measurable, and has been extensively discussed in the literature [9]. However, due to the ‘one and done’ behavior of ADR (i.e., an endoscopist may remove one adenoma and not check the rest of the colon carefully), there is growing consideration that a substitute may be needed, such as APC [9]. In addition, factors such as age, gender, obesity, and diabetes mellitus (DM) influence ADRs and should be in recommended targets [9].

While several studies in the US and European countries have established benchmarks for ADRs in both direct and FIT-based colonoscopy [10], developing nations have recently started implementing screening programs with no consensus on the optimal targets. Notably, these countries cannot simply adopt the screening goals of developed nations due to variations in the prevalence of CRC and adenoma across various regions worldwide. For example, several multicenter studies have reported different ADRs for direct colonoscopies in various countries, including 24.6% in Mexico [11], 15% in Egypt [12], 9.9% in Pakistan [13], and 12.8% in Iran [14]. As a result, each country should carefully evaluate its own situation, identify the risks and contributing factors, and set appropriate targets accordingly.

This study presents the primary results of the CRC screening program in Isfahan, Iran. The objective of this study was to assess the prevalence of adenomas and any variations in adenoma prevalence across cities and time periods. Additionally, this study aimed to explore the influence of demographic factors and comorbidities on adenoma detection rates.

## Methods

### Screening program

The Iranian Ministry of Health has been managing the CRC screening program in Isfahan Province since March 2017. As part of the program, the Cancer Registry of the Vice-Chancellor for Health at Isfahan University of Medical Sciences is responsible for implementing screening programs and gathering data in primary healthcare settings. In essence, healthcare providers assess risk factors for individuals between the ages of 50 and 70 who visit health centers, such as overt gastrointestinal (GI) bleeding, iron deficiency anemia, weight loss, history of inflammatory bowel disease, history of colorectal polyps, history of CRC, and first-degree family history of CRC. Individuals with any of the aforementioned risk factors are classified as high-risk and are immediately referred for colonoscopy. On the other hand, those without any risk factors are considered the average risk and are offered a single quantitative FIT. If the FIT results are positive, participants are referred for a colonoscopy. Furthermore, healthcare providers are responsible for follow-up care, including collecting colonoscopy and pathology reports and forwarding them to the Cancer Registry. All participants provided informed consent before inclusion in the study, following the ethical guidelines of the Declaration of Helsinki.

### Study design

This retrospective study involved all 21 cities in Isfahan Province (aged 50–70). This study utilized data from four sources of variables. The first source was the comprehensive e-health system (SIB in Persian), which provided information on patient age, gender, city, body mass index (BMI), smoking status, and the presence of DM, hypertension (HTN), dyslipidemia (DLP), and cardiovascular disease (CVD), including previous myocardial infarction or stroke. The second source of variables was the colonoscopy reports, which included the year of the colonoscopy, code of the colonoscopists, name of the colonoscopy center, bowel preparation quality, cecal intubation status, characteristics of polyps (such as number, size, and location), and presence of other pathologies (e.g., hemorrhoids, colitis, ulcers, fissures, diverticula, inflammation, and angiodysplasia). The third source was pathology reports, which provided information on the number and type of adenomas (including tubular adenoma, villus adenoma, sessile serrated polyp, traditional serrated adenoma, low-grade dysplasia, and high-grade dysplasia), as well as the presence of adenocarcinomas.

Individuals who participated in the first round of the CRC screening program between April 2017 and March 2022 and who were average-risk with a positive FIT result were included. Data were analyzed in five consecutive 12-month intervals (April–March) to represent complete screening years; therefore, tables display data up to 2021, corresponding to the last full year of observation. All colonoscopy and pathology reports from the Cancer Registry were reviewed to verify the accuracy of the available database. Out of the 5764 participants in the database, 2962 existing reports were identified. Individuals younger than 50 years (*n*=126), older than 70 years (*n*=88), at high-risk (*n*=473), and with no cecal intubation (*n*=66) were excluded. The final analysis included 2209 colonoscopy exams conducted by 54 endoscopists.

Although we excluded colonoscopies without cecal intubation, we decided to calculate the Cecal Intubation Rate (CIR) using the data before this exclusion. The CIR is defined as the percentage of colonoscopies in which the cecum is successfully reached by the endoscope. Additionally, we aimed to demonstrate whether colonoscopies without cecal intubation exhibit lower quality. The Medical Faculty at Isfahan University of Medical Sciences approved all the study materials and procedures.

### Measurements of detection rates

Proximal adenoma was defined as an adenoma located in the cecum, ascending colon, hepatic flexure, or transverse colon; distal adenoma was defined as an adenoma in the splenic flexure, descending colon, sigmoid colon, or rectum. We measured several quality indicators during the study, including:

1. PDR: The percentage of colonoscopies in which at least one polyp was identified and removed.
2. ADR: The percentage of colonoscopies with at least one histologically confirmed adenoma, excluding SSLs and CRCs.
3. AADR: The percentage of colonoscopies with at least one advanced adenoma (i.e. tubular adenomas > 1 cm, having high-grade dysplasia, tubulovillous, or villous characteristics).
4. prox-ADR: The percentage of colonoscopies with at least one histologically confirmed adenoma in the proximal side of the colon.
5. SDR: The percentage of colonoscopies with at least one histologically confirmed traditional serrated adenoma and sessile serrated adenomas/lesions with dysplasia detected.
6. APC: The average number of histologically confirmed adenomas detected per colonoscopy. (calculated by dividing the total number of adenomas detected by the total number of colonoscopies performed).

### Analysis

We excluded cardiovascular events from our analysis due to an unexpectedly high reported rate and a lack of documentation regarding the specific types of cardiovascular diseases. Smoking status was recorded; however, due to its low frequency (*n*=19) and potential reporting bias, it was not included in the analysis. In Iran, smoking is socially sensitive, and many individuals may not disclose it, particularly when accompanied by family members during medical visits. In the initial dataset, only 22 participants were identified as smokers. Categorical variables are presented as frequencies and were compared using the Pearson chi-square test of independence. Initially, binary logistic regression was applied to participants with complete baseline characteristics to identify variables associated with detection rates, resulting in unadjusted odds ratios (ORs) and 95% confidence intervals (CIs). Six variables (e.i, preparation, BMI, DM, HTN, DLP, and CVD) had substantial missing data and/or unadjusted *p*-values >0.1 for all detection rates. Consequently, we excluded them, as adjustments require variables with p-values less than 0.1 and complete information. Thus, only age, gender, year, colonoscopist, and center were included in multinomial logistic regression to calculate the adjusted OR (AOR) and 95% CI for 1922 individuals. To identify variables associated with APC, we initially used an independent t-test, followed by adjustment using multinomial linear regression with dummy variables. Bowel preparation methods were standardized according to recommended criteria [15]. To address missing values for the independent variables, binary logistic regression was employed to compare quality indicators between participants with available and missing data. A significance level of 0.05 was used in all tests. Data analysis was conducted using SPSS software (version 17.0.1.0).

## Results

Among 2,209 participants, 990 (44.82%) were male. The mean age of participants was 60.1 years (SD 5.8). Comorbidities such as DM and HTN were prevalent in 20.3% and 24.7% of the participants, respectively. Bowel preparation was documented in 67.4% of the reports, with a majority classified as good (70.8%). More details are presented in Table 1. The overall CIR was 97.1%. The odds of detecting polyps and adenomas were significantly higher in complete colonoscopies compared to incomplete ones (Table S1).

**Table 1:** Characteristics of included participants

Characteristic	Number (*n*)	Percentage (%)	Characteristic	Number (*n*)	Percentage (%)
Gender			Hypertension		
Male	990	44.82	Yes	321	14.53
Female	1219	55.18	No	426	19.28
Age (years)			Missed	1462	66.18
50–59	1092	49.43	Dyslipidemia		
60–69	1117	50.57	Yes	280	12.68
Center type			No	423	19.15
Public	1157	52.38	Missed	1506	68.18
Private	965	43.68	Cardiovascular disease		
Missed	87	3.94	Yes	42	1.90
Colonoscopist			No	721	32.64
Academic	517	23.40	Missed	1446	65.46
Non-academic	1610	72.88	Bowel preparation		
Missed	82	3.71	Good	1054	47.71
Year			Bad	435	19.69
2017	484	21.91	Missed	720	32.59
2018	608	27.52	Pathologies		
2019	663	30.01	Neoplasia	762	34.50
2020	325	14.71	Cancer	62	2.81
2021	129	5.84	Other pathologies	1183	53.55
BMI			- Hemorrhoids	959	43.41
< 25	994	45.00	- Diverticula	121	5.48
25–30	260	11.77	- Colitis	88	3.98
≥ 30	260	11.77	- Fissure	63	2.85
Missed	695	31.46	- Angiodysplasia	35	1.58
Diabetes			- Inflammation	28	1.27
Yes	159	7.20	- Ulcer	29	1.31
No	545	24.67	Missed	202	9.14
Missed	1505	68.13	Total	2209	100.00

### Detection indicators

At least one polyp, adenoma, and advanced adenoma were detected in 34.5%, 25.3%, and 10% of procedures, respectively. Distal adenomas accounted for 48%, proximal adenomas for 32%, and both proximal and distal adenomas for 19.3%, resulting in a prox-ADR of 13.0%. Additionally, SSLs were found in 1% of participants. The overall APC was 0.41. The relationship between patient characteristics included in multinomial logistic regression and APC and ADR is presented in Table 2.

**Table 2:** Relationship between patient characteristics included in multinomial logistic regression and APC and ADR

		APC			ADR		
Patient Characteristic	**Number**, *n* (%)	**Mean**	**Coefficient [95% CI]**	***P*** **-value**	**Percent**	**AOR [95% CI]**	***P*** **-value**
Gender							
Male	990 (44.8)	0.54	0.117 [0.077, 0.156]	0.000*	0.54	1.76 (1.38–2.24)	0.000*
Female	1219 (55.2)	0.32			0.32		
Age (years)							
50–59	1092 (49.4)	0.41	0.005 [0.002, 0.009]	0.005*	0.41		
60–69	1117 (50.6)	0.46			0.46	1.14 (0.97–1.34)	0.005*
Center							
Public	1157 (52.2)	0.46	0.032 [-0.010, 0.074]	0.139	0.46	1.03 (0.90–1.16)	0.139
Private	965 (43.7)	0.35			0.35		
Colonoscopist							
Academic	517 (23.4)	0.47	0.046 [-0.001, 0.093]	0.056	0.47	1.05 (0.91–1.20)	0.056
Non-academic	1610 (72.9)	0.39			0.39		
Year							
2017	484 (21.9)	0.42	-0.007 [-0.062, 0.048]	0.806	0.42	0.99 (0.95–1.03)	0.806
2018	608 (27.5)	0.40	-0.013 [-0.064, 0.038]	0.609	0.40	0.99 (0.96–1.03)	0.609
2019	663 (30.0)	0.50	Ref		0.50	Ref	—
2020	325 (14.7)	0.30	-0.067 [-0.132, -0.003]	0.039*	0.30	0.93 (0.87–0.99)	0.039*
2021	129 (5.8)	0.33	-0.079 [-0.168, 0.010]	0.081	0.33	0.92 (0.84–1.01)	0.081

*APC* Adenoma per colonoscopy, *ADR* Adenoma detection rate, *Significance at 0.05

### Factors influencing

#### Gender

The ADR was significantly greater in men (31.2%) than in women (20.4%) (*p* < 0.001, phi=0.12). The estimated risk of adenoma was almost double in men compared to women (AOR 1.84; 95% CI, 1.49–2.69). Additionally, 41.2% of men and 29.0% of women had polyps (AOR 2.82; 95% CI, 1.50–2.20). Similar trends were noted in AADR (AOR 2.04), Prox-ADR (AOR 1.72), and SDR (AOR 3.40). Gender significantly impacted APC, with males showing a greater APC of 0.54 compared to females at 0.32, remaining significant after adjustment (Beta 0.12; 95% CI, 0.08, 0.16; *p* < 0.001). Adequate bowel preparation was not significantly different between men and women (70.9 vs. 70.6) (Table S2).

#### Age

There was a significant difference in PDR between individuals aged 60–69 (39.4%) and those aged 50–59 (33.5%) (AOR 1.33; 95% CI, 1.10–1.62; *p* = 0.003). Similarly, ADR differed between ages 60–69 (28.8%) and 50–59 (24.5%) (AOR 1.28; 95% CI, 1.04–1.58; *p* = 0.02). Prox-ADR also varied between the groups (AOR 1.31; 95% CI, 1.01–1.71; *p* = 0.04). No significant differences were found for AADR and SDR (*p* = 0.48). Patients aged 60–69 showed a greater APC of 0.46 compared to those aged 50–59 (0.41), remaining significant after adjustment (*p* = 0.005). Adequate bowel preparation was not significantly different (69.0 vs. 70.3) (Table S3).

#### Year of colonoscopy

Trends across various years revealed differences in PDR (*p*=0.001), with significant differences between specific pairs such as 2017 and 2020, and 2019 and 2020. While ADR showed differences (*p*=0.02), further analysis found no specific pairwise significance. AADR, Prox-ADR, and SDR did not significantly differ (*p*=0.15, 0.25, and 0.33, respectively). A trend in APC over the years was observed, with the only significant difference noted in 2020 compared to 2019 (0.30 vs. 0.50). There was a significant improvement in good bowel preparation over the years. The rate increased from 62.7% in 2017 to 84.5% in 2019, although it slightly decreased to 73.3% in 2021 (Table S4).

#### Center type

Polyps were significantly more frequently found in public centers compared to private centers (36.9% vs. 30.7%), even after adjusting (AOR 1.28; 95% CI, 1.04–1.57; *p* = 0.018). However, this was not the case for adenomas, advanced adenomas, proximal adenomas, and SSLs (*p* > 0.1). While APC appeared higher in public centers (0.46 vs. 0.35), these differences were not significant after adjustment (*p*=0.14). Private centers had a significantly greater rate of good bowel preparation (79.3% vs. 64.0%) (Table S5).

#### Colonoscopist

There was a significant increase in detection rates in the academic group: PDR increased by 1.34-fold, ADR by 1.27-fold, AADR by 1.47-fold, prox-ADR by 1.12-fold, and SDR by 2.93-fold. Only PDR, AADR, and SDR were significant. APC appeared higher in academic colonoscopists (0.47 vs. 0.39), but not significant after adjustment (*p*=0.06). Academic colonoscopists achieved a significantly higher rate of good bowel preparation (82.1% vs. 34.5%) (Table S6).

#### BMI

In the group of 1514 individuals with BMI data, the ADR was 25.2 for normal-weight patients, 30.8 for overweight, and 27.7 for obese patients, with no statistically significant differences. Post-hoc analysis also showed no significance. APC was higher among individuals with a BMI > 25 (0.48 vs. 0.40). Good preparation was slightly greater in overweight individuals compared to normal weight (72.8% vs. 70.4%), but significantly lower in those with obesity (58.5%) (Table S7).

#### Comorbidities

Data for other comorbidities were available for approximately 700 participants. The DR was greater in those with DM (34 vs. 27.7), and lower in those with HTN (26.2 vs. 30.3) and DLP (27.0 vs. 30.4), but not statistically significant (*p*=0.12, 0.21, and 0.31). The relative risk for diabetes was 1.06. A similar trend was observed for other detection rates, except in two instances: Prox-ADR was slightly greater in HTN (16.8 vs. 14.8; *p*=0.45) and AADR was slightly greater in DLP (13.5 vs. 12.9; *p*=0.82). ADR for participants with or without CVD was 28.6. Other detection rates, except for SDR, were lower in participants with a positive history, but not significantly (*p* > 0.1). APC was only greater among individuals with DM (0.44 vs. 0.62) and a history of CVD (0.47 vs. 0.43). Good preparation rates were slightly lower in patients with DM, DLP, and CVD, and slightly greater in those with HTN, but differences were not significant (Table S8-S11).

#### Bowel preparation

Bowel preparation was documented in only 67.4% of the reports. Reporting methods included BBPS (32.04%), adequate (19.41%), good (15.52%), poor (8.73%), fair (6.47%), and suboptimal (6.03%). Other categories, each constituting less than 3% of the total, included acceptable, inadequate, bad, not good, moderate, optimum, not fair, excellent, and very poor. These methods were consolidated into a general classification: bad (29.2%) and good (70.8%), with no significant quality variation identified. APC remained at 0.41 for both patients with good and poor bowel preparation (Table S12).

Finally, the factors significantly influencing ADR were gender, age, and the year of colonoscopy. For PDR, the significant factors were gender, age, year of colonoscopy, center, and type of colonoscopist. AADR was significantly influenced by gender and the type of colonoscopist. Prox-ADR was influenced by gender and age. SDR was affected by gender and the type of colonoscopist. APC was influenced by gender, age, and year of colonoscopy. Adequate preparation was significantly associated with the private center, academic colonoscopist, year of colonoscopy, and BMI.

### Impact of missing data

Missing data were substantial for BMI (31.5%), DM (68.1%), HTN (67.9%), DLP (67.9%), CVD (65.5%), and bowel preparation (32.6%). Center and colonoscopist had lower missing rates (3.9% and 3.7%, respectively). Missing BMI data significantly reduced PDR, ADR, AADR and prox-ADR. Missing data for DM, HTN, DLP, and CVD significantly lowered most detection rates except SDR. Missing center data increased PDR (44.8% vs. 34.1%, *p*=0.039), while missing colonoscopist data showed no significant impact. Missing bowel preparation data had no significant effect on detection rates (Table S13–S20).

## Discussion

This study presents the primary results of the CRC screening program in Isfahan, Iran, from 2012 to 2019. These findings offer insights into the quality of colonoscopies performed in a country with a newly established screening program.

The prevalence of adenoma after a positive FIT was 25.3%, lower than ADRs reported in European studies using FIT (43.5% to 51.5%) or gFOBT (35% to 47%) [16–19]. This finding is consistent with results from developing countries with lower CRC prevalence, such as 14.3% (Malaysia; 49 individuals), 17.7% (Iran; 113 individuals), and 26.9% (Indonesia; 221 individuals) [20–22]. As extensively observed, ADR in direct screening programs tends to be lower compared to FIT-based screening colonoscopies, typically from 20% to 25% [23, 24]. However, even these figures notably diminish in developing countries: Mexico reports an ADR of 24.6% [11], Egypt 15%, Pakistan 9.9% [12, 13] and India between 4.35% and 6.7% [25–27]. In Iran, a study of 531 individuals aged 15 to 85 years who underwent colonoscopy for various reasons revealed a PDR of 23.5%, an ADR of 12.8%, and a CRC rate of 1.5% [14]. Similarly, among 1676 asymptomatic average- and high-risk participants aged 50–70 years who underwent direct screening colonoscopy, the PDR and ADR were 22.7% and 13.5%, respectively [28]. While this variation may stem from underestimation due to missed lesions, significant disparities are primarily attributed to differing prevalence rates of CRC and adenomas within populations [2]. In Iran, a developing country, the incidence of CRC is approximately half that observed in the United States [3]. Interestingly, these discrepancies are frequently linked to environmental factors, particularly dietary habits, rather than racial differences [29].

Our overall PDR (34.5%) was lower than in Western populations [8], but comparable to those from Pakistan (7.9–11.3%) and India (10.2–12.7%) [25, 27, 30, 31]. Similar to previous studies, adenoma detection was higher in men and increased with age [8, 17, 24, 32, 33].

In our study, the SDR was 1%, consistent with the wide variability reported in previous studies. A meta-analysis on the prevalence of SSLs and their association with synchronous colorectal advanced neoplasia found that reported SSL prevalence ranged from 0.038% to 20.23%, with a pooled estimate of 2.7% [34]. This variation likely reflects a gap between the true prevalence of SSLs and their reported detection rates, which differ substantially across centers [35]. Many SSLs are missed during colonoscopy because of their subtle, flat appearance and limited endoscopic recognition. Factors such as lack of gastroenterology speciality training, fewer years of experience (≤ 5 years), and lower procedure volumes are independently associated with reduced serrated polyp detection [35]. Even when detected, SSLs may not be correctly identified or confirmed histologically due to incomplete sampling or diagnostic variability among pathologists, partly related to evolving histopathologic criteria [36].

According to the American Society for Gastrointestinal Endoscopy (ASGE)/American College of Gastroenterology (ACG), smoking, obesity, and DM influence adenoma prevalence. However, adjusting the target ADR for these factors is not currently recommended [8].

In this study, ADR was higher in DM, though not significantly. Conversely, a recent meta-analysis revealed that DM significantly elevates the risk of adenomas and patients with DM show higher risk of adenoma [37]. This may be attributed to insulin resistance promoting tumor growth, and slower bowel transit times increasing carcinogen exposure [38]. However, the meta-analysis couldn’t control for factors such as age, ethnicity, inactivity, aspirin use, obesity, family history, and menopausal status due to data limitations, which are common risk factors for both DM and adenoma [39].

Research indicates a connection between higher BMI and an increased risk of adenomas [40]. Participants with overweight and obesity had a 44% and 42% higher risk of adenomas, respectively, according to a meta-analysis of 17 articles. Six of these articles showed a positive but not significant correlation [41], which mirrors our findings. This trend of increasing ADR in participants with overweight, followed by a decrease in those with obesity, can be explained by our observation of decreasing adequate bowel preparation in participants with obesity. This worsens patient intolerance and poses challenges for physicians. Other reasons include population-based factors such as small sample size, racial or socioeconomic differences, and the recent adoption of a Western lifestyle in our country, which has increased obesity but has yet to significantly impact neoplasms incidence.

While not extensively assessed, certain studies have indicated a correlation between detection rates and medical centers characteristics [17]. Our research found that among all detection rates, only PDR was higher in public settings compared to private ones. Similarly, A Korean study with 1,064 participants found significantly higher PDR in community hospitals than in nonhospital facilities [42]. While a US study found no relationship [43], a large Spanish study revealed higher ADR, AADR, and CIR in non-academic hospitals [44]. Additionally, detection rates were generally higher among academic colonoscopists, significantly so for PDR and AADR. This aligns with findings of fewer interval cancers [45] and higher proximal serrated PDRs in academic settings [46], but contradicts findings of no lower ADRs in nonacademic settings [47]. Further research is needed to clarify the factors underlying the differences observed.

Inadequate bowel preparation was detected in 29.8%, higher than rates reported in Pakistan at 12% [30], the USA at 21.2% [48], but lower than other studies reporting 30-44.2% [49–51]. As per recommendations from ASGE/ ACG, an inadequate bowel preparation rate exceeding 15% is concerning due to its association with unsuccessful screens, the need for repeat procedures, and consequent costs [8]. This issue is exacerbated in non-safety net healthcare systems, where patients may be lost to follow-up [51]. Lower rates of adequate bowel preparation might stem from imperfect reporting methods, medication non-compliance, or misunderstanding instructions. Different protocols were employed across our centers, and data on specific instructions is unavailable. Therefore, it is imperative to review bowel preparation protocols, including patient education, choice of purgative, and administration methods such as the split-dose approach [9].

Our findings showed no correlation between PDR, ADR, AADR, and Prox-ADR with bowel preparation quality. This should be interpreted with caution due to limitations like using various and non-validated reporting methods, which allowed categorizing into only two groups. As mentioned in previous research, the diverse measures used by clinicians cause confusion and difficulty in comparisons [15]. Though unexpected, many studies question the linear relationship between detection rates and preparation. For instance, ADRs were higher in high- or intermediate-quality preps compared to low-quality ones, but not significantly different between intermediate and high-quality preps [52]. Additionally, while oral simethicone significantly increased preparation quality, it did not affect ADR, PDR, or CIR except among clinicians with imperfect performance [53]. Notably, segmental ADR and SDR decrease as preparation scores increase, particularly in patients with excellent preparation [54]. This phenomenon can be explained by poor preparation makes the colonoscopist pay better attention, avoiding a false sense of confidence. Additionally, colonoscopists may rate preparation as excellent when no polyps are found [55]. So, validated scales can help clarify colonoscopy performance.

In this study, private centers demonstrated better preparation, possibly due to superior resources, standardized protocols for patient education and support, and shorter wait times for procedures. Another theory suggests participants may follow instructions better, or endoscopists may rate colon preparation more favorably in advanced settings. Additionally, academic colonoscopists consistently rated preparation higher, warranting investigation, as it may be due to better knowledge on preparation scales, patient adherence, or patient education skills. Besides, patient populations at each site, and differences in patients’ understanding are also matters. Studies have shown varying results across different countries: in Spain, adequate preparation was higher in non-academic hospitals [44]; in the US, good preparation was highest in private practice groups, followed by community-based hospitals, and then tertiary care hospitals [43]. In Korea, there was no significant difference in preparation between community hospitals and non-hospital facilities [42]. We advocate for further research to examine workload and cost considerations, as these aspects are important in various settings and remain under-evaluated.

The results support studies indicating gender and age are not independent risk factors for bowel preparation [51, 56], but contradict others [48, 49, 51]. These discrepancies are probably due to the retrospective nature of this study, which did not consider compliance with preparation instructions, narcotic use, drug history, changes in bowel habits, history of colonoscopy, differences in socioeconomic status, and definitions of adequate preparation [49–51].

Adequate bowel preparation tends to decrease as BMI increases, a trend observed in our population and others [49, 50]; each unit rise in BMI is associated with a 2.1% increase in inadequate composite scores [51]. This is particularly important due to the increased incidence of colon cancer among obese patients [2]. However, some studies show contradictory results [48, 56], possibly due to reasons mentioned earlier [49–51]. Additionally, the relationship of DM with intestinal enteropathy and gastroparesis is known to cause constipation [57] and poorer preparation, anticipatedly. While several studies confirmed this [48, 51], this result supported the trend but not significantly. This may be due to a small sample size or other aforementioned conflicting factors [49–51].

The observed CIR was 97%, meeting the ASGE/ACG recommendation of ≥ 95% [8] and surpassing the rates reported in several studies [30, 47]. In incomplete procedures, detection rates were notably lower, suggesting that procedures without cecal intubations may fail to identify the anticipated number of polyps. Successful cecal intubation significantly enhances colonoscopy efficacy in detecting potentially significant lesions.

One of the strengths of this report is its multi-center design and the inclusion of several factors influencing neoplasia yield, such as patient-related variables like BMI, past medical history, and bowel preparation. Our study does have some limitations.

We were unable to evaluate endoscopist-related factors (e.g., endoscopist experience, withdrawal time and technique), endoscope-related factors (e.g., instrument generation), or center-related factors (e.g., dedicated screening sessions) because our study relied on routine clinical data. Recording these parameters was neither customary nor mandatory during the study period. Inconsistent reporting, limited resources, and the lack of standardized electronic records further contributed to this gap.

Additionally, we did not include other important factors like diet and lifestyle. The sample was not randomly selected, so the findings must be interpreted with caution as the data were not population-based. This study focused solely on one province; therefore, the results may not be entirely representative of the whole country. Moreover, our database relied on clinical data rather than analytical data, making it susceptible to human error and misclassification bias. Another important limitation of our study is that missing data were substantial for many variables, and our analysis showed that this missingness affected detection rates and APC. Therefore, these results should be interpreted with extreme caution. A further limitation is the underreporting of smoking. Cultural factors may lead participants to conceal smoking habits, especially when visiting the clinic with family. Consequently, the number of smokers in our study was very low (*n* = 19), limiting the ability to assess its effect on neoplasia risk.

## Conclusion

The study provides valuable insights into the adenoma detection rates and factors influencing colonoscopy quality within the CRC screening program in Isfahan, Iran. The findings highlight the significance of localized benchmarks, standardized training, and tailored bowel preparation protocols to enhance the effectiveness of CRC screening programs. Continued efforts to improve colonoscopy quality indicators will contribute to early detection and prevention of colorectal cancer, ultimately reducing its incidence and mortality in developing countries.

We recommend that physicians consider men, older individuals, and those with diabetes or obesity more often. Regarding bowel preparation, standardized training on scoring using uniform scales is essential. We propose reevaluating the type and dosage of bowel preparation, tailoring regimens to individual patient needs, especially obese patients, and enhancing patient education, particularly in public healthcare centers and among nonacademic practitioners. Finally, we suggest enhancing quality control systems. Given that ADR as a quality indicator is dependent on background epidemiology, implementing a dynamic living standard point based on extensive colonoscopy data is crucial. This would provide updated cut-offs and enable continuous auditing of the process based on new data.

Future studies should incorporate endoscopist-related factors (e.g., withdrawal time), endoscope-related factors, and endoscopy center-related factors to enhance the assessment of colonoscopy quality.

## Supplementary Information

Supplementary Material 1. Table S1- Relationship between cecal intubation and PDR, ADR, AADR, SDR, and APC. Table S2- Relationship between gender and PDR, ADR, AADR, SDR, and APC. Table S3- Relationship between age groups and PDR, ADR, AADR, SDR, and APC. Table S4- A- Trends of the year in relation to PDR, ADR, AADR, SDR, and APC. B- Adjusted OR (95% CI) of the years in relation to PDR, ADR, AADR, and SDR. Table S5- Relationship between type of colonoscopy centers and PDR, ADR, AADR, SDR, and APC. Table S6- Relationship between being an academic colonoscopist and PDR, ADR, AADR, SDR, and APC. Table S7- Relationship between BMI and PDR, ADR, AADR, SDR, and APC. Table S8- Relationship between diabetes and PDR, ADR, AADR, SDR, and APC. Table S9- Relationship between hypertension and PDR, ADR, AADR, SDR, and APC. Table S10- Relationship between hyperlipidemia and PDR, ADR, AADR, SDR, and APC. Table S11- Relationship between CVD and PDR, ADR, AADR, SDR, and APC. Table S12- Relationship between bowel preparation and PDR, ADR, AADR, SDR, and APC. Table S13- Relationship between center and PDR, ADR, AADR, SDR, and APC; missing data. Table S14- Relationship between colonoscopist and PDR, ADR, AADR, SDR, and APC; missing data. Table S15 - Relationship between obesity and PDR, ADR, AADR, SDR, and APC; missing data. Table S16- Relationship between diabetes and PDR, ADR, AADR, SDR, and APC; missing data. Table S17- Relationship between hypertension and PDR, ADR, AADR, SDR, and APC; missing data. Table S18- Relationship between hyperlipidemia and PDR, ADR, AADR, SDR, and APC; missing data. Table S19- Relationship between CVA and PDR, ADR, AADR, SDR, and APC; missing data. Table S20- Relationship between bowel preparation and PDR, ADR, AADR, SDR, and APC; missing data.

## Data Availability

All data generated and analyzed during this study are included in this published article.
