# Supplementary material for: Evaluating quality indicators in colorectal cancer screening via fecal immunochemical tests: a five-year study from a developing country
Source: BMC Gastroenterol. 2025 Dec 30;26:82. doi: 10.1186/s12876-025-04527-1 (PMC12860085; doi:10.1186/s12876-025-04527-1)
Supplement: Supplementary file 1 — Supplementary Material 1. Table S1- Relationship between cecal intubation and PDR, ADR, AADR, SDR, and APC. Table S2- Relationship between gender and PDR, ADR, AADR, SDR, and APC. Table S3- Relationship between age groups and PDR, ADR, AADR, SDR, and APC. Table S4- A- Trends of the year in relation to PDR, ADR, AADR, SDR, and APC. B- Adjusted OR (95% CI) of the years in relation to PDR, ADR, AADR, and SDR. Table S5- Relationship between type of colonoscopy centers and PDR, ADR, AADR, SDR, and APC. Table S6- Relationship between being an academic colonoscopist and PDR, ADR, AADR, SDR, and APC. Table S7- Relationship between BMI and PDR, ADR, AADR, SDR, and APC. Table S8- Relationship between diabetes and PDR, ADR, AADR, SDR, and APC. Table S9- Relationship between hypertension and PDR, ADR, AADR, SDR, and APC. Table S10- Relationship between hyperlipidemia and PDR, ADR, AADR, SDR, and APC. Table S11- Relationship between CVD and PDR, ADR, AADR, SDR, and APC. Table S12- Relationship between bowel preparation and PDR, ADR, AADR, SDR, and APC. Table S13- Relationship between center and PDR, ADR, AADR, SDR, and APC; missing data. Table S14- Relationship between colonoscopist and PDR, ADR, AADR, SDR, and APC; missing data. Table S15 - Relationship between obesity and PDR, ADR, AADR, SDR, and APC; missing data. Table S16- Relationship between diabetes and PDR, ADR, AADR, SDR, and APC; missing data. Table S17- Relationship between hypertension and PDR, ADR, AADR, SDR, and APC; missing data. Table S18- Relationship between hyperlipidemia and PDR, ADR, AADR, SDR, and APC; missing data. Table S19- Relationship between CVA and PDR, ADR, AADR, SDR, and APC; missing data. Table S20- Relationship between bowel preparation and PDR, ADR, AADR, SDR, and APC; missing data. [file 12876_2025_4527_MOESM1_ESM.docx]

**Supplementary data**

For all tables:

PDA = Polyp detection rate, ADR = Adenoma detection rate, AADR = Advanced adenoma detection rate, SDR = Serrated adenoma detection rate, APC = Adenoma per colonoscopy, *Significance at the 0.05 level.

**Table S1-** Relationship between cecal intubation and PDR, ADR, AADR, SDR, and APC.

|  | With cecal intubation | Without cecal intubation | P-value, Phi | OR (95% CI)  (with cecal intubation: ref) |
| --- | --- | --- | --- | --- |
| Number, n (%) | 2209 (97.1) | 66 (2.9) |  |  |
| PDR, % | 34.5 | 21.2 | 0.025*, -0.47 | 1.956 (1.077 - 3.552) |
| ADR, % | 25.3 | 15.2 | 0.062 | 1.893 (0.959 - 3.735) |
| AADR, % | 10.0 | 9.1 | 0.79 | 1.117 (0.477 - 2.616) |
| Prox-ADR, % | 13.0 | 7.6 | 0.19 | 1.829 (0.729 - 4.590) |
| SDR, % | 1.0 | 0.0 | 0.41 | - |
| APC, n | 0.41 | 0.18 | <0.001* |  |

**Detection rates**

**Table S2**- Relationship between ***gender*** and PDR, ADR, AADR, SDR, and APC.

|  | **All** | **Male** | **Female** | **P-value ^a.^ Phi** | **Unadjusted OR/ Beta Coeffient (95% CI)**  **(Female: ref)** | **Adjusted OR/ Beta Coeffient (95% CI), P-value ^b^**  **(Female: ref)** |
| --- | --- | --- | --- | --- | --- | --- |
| **Number, n (%)** | **2209** | **990 (44.8)** | **1219 (55.2)** |  |  |  |
| **PDR, %** | 34.5 | 41.2 | 29.0 | 0.000, 0.127 | 1.713 (1.435-2.045) | 1.819 (1.502 - 2.203), 0.000 |
| **ADR, %** | 25.3 | 31.2 | 20.4 | 0.000, 0.123 | 1.768 (1.457-2.145) | 1.844 (1.499 - 2.269), 0.000 |
| **AADR, %** | 10.0 | 13.1 | 7.5 | 0.000, 0.092 | 1.852 (1.398-2.454) | 2.041 (1.514, 2.749), 0.000 |
| **Prox-ADR, %** | 13.0 | 16.6 | 10.2 | 0.000, 0.094 | 1.753 (1.365-2.252) | 1.719 (1.319, 2.241), 0.000 |
| **SDR, %** | 1.0 | 1.5 | 0.6 | 0.027, 0.047 | 2.664 (1.082-6.559) | 3.401 (1.300, 8.898), 0.013 |
| **APC, n** | 0.41 | 0.54 | 0.32 | 0.000 |  | 0.117 [0.077, 0.156], 0.000* |

^a^ Before adjustment, by Chi-square test.

^b^ After adjustment, by multinomial logistic regression/ linear regression.

**Table S3**- Relationship between ***age*** groups and PDR, ADR, AADR, SDR, and APC.

|  | **All** | **60-69 years** | **50-59 years** | **P-value ^a,^ Phi** | **Unadjusted OR (95% CI) / Beta Coeffient**  **(50-59 years: ref)** | **Adjusted OR/ Beta Coeffient (95% CI), P-value ^b^**  **(50-59 years: ref)** |
| --- | --- | --- | --- | --- | --- | --- |
| **Number, n (%)** | **2026** | **1117 (50.6)** | **1092 (49.4)** |  |  |  |
| **PDR, %** | 36.4 | 37.2 | 31.9 | 0.009, 0.06 | 1.289 (1.075-1.545) | 1.332 (1.100-1.613), 0.003 |
| **ADR, %** | 26.6 | 27.1 | 23.5 | 0.027, 0.05 | 1.249 (1.025-1.522) | 1.282 (1.042, 1.577), .019 |
| **AADR, %** | 10.6 | 10.2 | 9.9 | 0.859 | 1.062 (0.800-1.409) | 1.111 (0.828, 1.491), .482 |
| **Prox-ADR, %** | 13.8 | 14.4 | 11.7 | 0.064, 0.04 | 1.300 (1.009-1.675) | 1.314 (1.008, 1.713), .043 |
| **SDR, %** | 1.1 | 1.2 | 0.8 | 0.363 | 1.488 (0.633-3.498) | 1.370 (0.567, 3.309), .484 |
| **APC, n** | 0.43 | 0.46 | 0.41 | 0.191 |  | 0.005 [0.002, 0.009], 0.005* |

^a^ Before adjustment, by Chi-square test.

^b^ After adjustment, by multinomial logistic regression/ linear regression.

**Table S4- A**- Trends of the ***year*** in relation to PDR, ADR, AADR, SDR, and APC.

|  | **All** | **2017** | **2018** | **2019** | **2020** | **2021** | **P-value ^a^, Phi** | **Adjusted OR/ Beta (95% CI), P-value ^b^** | | | | |
| --- | --- | --- | --- | --- | --- | --- | --- | --- | --- | --- | --- | --- |
|  |  |  |  |  |  |  |  | **2017** | **2018** | **2019** | **2020** | 2021 |
| **Number, n (%)** | 2209 | 484 (21.9) | 608 (27.5) | 663 (30.0) | 325 (14.7) | 129 (5.8) |  |  |  |  |  |  |
| **PDR, %** | 34.5 | 37.4 | 33.7 | 38.3 | 26.2 | 28.7 | 0.001, 0.091 | ref, .013 | .859 (0.655, 1.125), .270 | .975 (0.749, 1.269), .849 | .628 (0.446, 0.885), .008 | .573 (0.361, 0.909), .018 |
| **ADR, %** | 25.3 | 26.4 | 25.2 | 28.4 | 19.1 | 20.9 | 0.021, 0.072 | ref, .149 | 0.967 (0.722, 1.296), .824 | 1.034 (0.778, 1.375), .816 | 0.710 (0.488, 1.033), .073 | 0.676 (0.409, 1.117), .126 |
| **AADR, %** | 10 | 11 | 11.2 | 10.6 | 7.1 | 6.2 | 0.148 | Ref, .100 | 1.013 (0.682, 1.506), .949 | 0.865 (0.581, 1.289), .477 | 0.632 (0.366, 1.092), .100 | 0.377 (0.156, 0.909), .030 |
| **Prox-ADR, %** | 13 | 13.6 | 13 | 14.6 | 9.5 | 11.6 | 0.251 | Ref, .729 | 0.957 (0.660, 1.385), .814 | 0.971 (0.676, 1.395), .874 | 0.751 (0.465, 1.214), .242 | 0.765 (0.408, 1.437), .406 |
| **SDR, %** | 1 | 1.7 | 1 | 0.6 | 1.2 | 0 | 0.327 | Ref, .664 | 0.567 (0.181, 1.770), .328 | 0.412 (0.121, 1.403), .156 | 0.841 (0.241, 2.940), .787 | .000 (.000, .000), .996 |
| **APC, n** | 0.41 | 0.42 | 0.4 | 0.5 | 0.3 | 0.33 | 0.017* | -0.007  [-0.062, 0.048],  0.806 | -0.013  [-0.064, 0.038],  0.609 | Ref | -0.067  [-0.132, -0.003],  0.039* | -0.079  [-0.168, 0.010],  0.081 |

^a^ Before adjustment, by Chi-square test/ an independent T-test.

^b^ After adjustment, by multinomial logistic regression/ linear regression.

**Table S4-B-** Adjusted OR (95% CI) of the ***years*** in relation to PDR, ADR, AADR, and SDR.

|  | **Adjusted OR (95% CI), P-value** | | | | |
| --- | --- | --- | --- | --- | --- |
|  | **PDR** | **ADR** | **AADR** | **Prox-ADR** | **SDR** |
| **2017** | ref, .013 | ref, .149 | Ref, .100 | Ref, .729 | Ref, .664 |
| **2018** | .859 (0.655, 1.125), .270 | 0.967 (0.722, 1.296), .824 | 1.013 (0.682, 1.506), .949 | 0.957 (0.660, 1.385), .814 | 0.567 (0.181, 1.770), .328 |
| **2019** | .975 (0.749, 1.269), .849 | 1.034 (0.778, 1.375), .816 | 0.865 (0.581, 1.289), .477 | 0.971 (0.676, 1.395), .874 | 0.412 (0.121, 1.403), .156 |
| **2020** | .628 (0.446, 0.885), .008 | 0.710 (0.488, 1.033), .073 | 0.632 (0.366, 1.092), .100 | 0.751 (0.465, 1.214), .242 | 0.841 (0.241, 2.940), .787 |
| **2021** | .573 (0.361, 0.909), .018 | 0.676 (0.409, 1.117), .126 | 0.377 (0.156, 0.909), .030 | 0.765 (0.408, 1.437), .406 | .000 ( .000, .000), .996 |

**Table S5**- Relationship between type of colonoscopy ***centers*** and PDR, ADR, AADR, SDR, and APC.

|  | **All** | **Public** | **Private** | **P-value ^a^** | **Unadjusted OR/ Beta Coeffient (95% CI)**  **(Private: ref)** | **Adjusted OR/ Beta Coeffient (95% CI), P-value ^b^**  **(Private: ref)** |
| --- | --- | --- | --- | --- | --- | --- |
| **Number, n (%)** | **2122** | **1157 (52.3)** | **965 (43.7)** |  |  |  |
| **PDR, %** | 34.1 | 36.9 | 30.7 | 0.003, 0.065 | 1.322 (1.102-1.585) | 1.280 (1.043-1.570), 0.018 |
| **ADR, %** | 25.1 | 26.8 | 23.1 | 0.051 | 1.218 (0.999-1.485) | 1.175 (0.942, 1.466), .152 |
| **AADR, %** | 10.1 | 10.5 | 9.5 | 0.441 | 1.119 (0.841-1.488) | 1.084 (0.790, 1.487), .618 |
| **Prox-ADR, %** | 12.9 | 13.7 | 12.0 | 0.263 | 1.158 (0.896-1.496) | 1.145 (0.864, 1.518), .346 |
| **SDR, %** | 1.0 | 1.3 | 0.6 | 0.118 | 2.099 (0.811-5.432) | 1.481 (0.527, 4.163), .456 |
| **APC, n** | 0.41 | 0.46 | 0.35 | 0.009 |  | 0.032 [-0.010, 0.074], 0.139 |

^a^ Before adjustment, by Chi-square test.

^b^ After adjustment, by multinomial logistic regression/ linear regression.

**Table S6**- Relationship between being an ***academic*** colonoscopist and PDR, ADR, AADR, SDR, and APC.

|  | **All** | **academic** | **Not-academic** | **P-value ^a^** | **Unadjusted OR/ Beta Coeffient (95% CI)**  **(Not-academic: ref)** | **Adjusted OR/ Beta Coeffient (95% CI), P-value^b^**  **(Not-academic: ref)** |
| --- | --- | --- | --- | --- | --- | --- |
| **Number, n (%)** | **2127** | **517 (24.3)** | **1610 (75.7)** |  |  |  |
| **PDR, %** | 34.2 | 41.0 | 32.0 | 0.000, 0.081 | 1.474 (1.202-1.807) | 1.344 (1.072-1.685), 0.010 |
| **ADR, %** | 25.1 | 29.2 | 23.8 | 0.013, 0.054 | 1.322 (1.059-1.650) | 1.274 (0.998, 1.625), .052 |
| **AADR, %** | 10.1 | 13.0 | 9.1 | 0.012, 0.055 | 1.482 (1.089-2.016) | 1.472 (1.051-2.062), 0.025 |
| **Prox-ADR, %** | 12.8 | 14.5 | 12.3 | 0.191 | 1.210 (0.909-1.611) | 1.125 (0.824, 1.537), .458 |
| **SDR, %** | 1.0 | 2.3 | 0.6 | 0.001, 0.072 | 3.802 (1.633-8.852) | 2.932 (1.155, 7.444), .024 |
| **APC, n** | 0.41 | 0.47 | 0.39 | 0.092 |  | 0.046 [-0.001, 0.093], 0.056 |

^a^ Before adjustment, by Chi-square test.

^b^ After adjustment, by multinomial logistic regression/ linear regression.

**Table S7**- Relationship between ***BMI*** and PDR, ADR, AADR, SDR, and APC.

|  | **All** | **BMI< 25** | **25 ≤ BMI< 30** | **BMI≥ 30** | **P-value** | **Post-hoc** |
| --- | --- | --- | --- | --- | --- | --- |
| **Number, n (%)** | **2209** | **994 (45.0)** | **260 (11.8)** | **260 (11.8)** |  |  |
| **PDR, %** | 36.0 | 35.2 | 40.0 | 35.0 | 0.335 | Not significant. |
| **ADR, %** | 26.6 | 25.2 | 30.8 | 27.7 | 0.170 | Not significant. |
| **AADR, %** | 10.9 | 9.8 | 11.9 | 14.2 | 0.101 | Not significant. |
| **Prox-ADR, %** | 14.1 | 13.2 | 16.2 | 15.4 | 0.376 | Not significant. |
| **SDR, %** | 1.1 | 1.3 | 1.5 | 0.4 | 0.418 | Not significant. |
| **APC, n** | 0.43 | 0.40 | 0.48 | 0.48 | 0.296 | With turkey: Not sig. |

**Table S8**- Relationship between diabetes and PDR, ADR, AADR, SDR, and APC.

|  | **All** | **Diabetes** | **Non diabetes** | **P-value** | **Unadjusted OR (95% CI)**  **(Non-diabetics: ref)** | **RR** |
| --- | --- | --- | --- | --- | --- | --- |
| **Number, n (%)** | **704** | **159 (22.6)** | **545 (77.4)** |  |  |  |
| **PDR, %** | 39.3 | 43.4 | 38.2 | 0.235 | 1.242 (0.868-1.777) | 1.05 |
| **ADR, %** | 29.1 | 34 | 27.7 | 0.127 | 1.342 (0.919-1.958) | 1.06 |
| **AADR, %** | 12.8 | 15.7 | 11.9 | 0.207 | 1.378 (0.836-2.270) | 1.07 |
| **Prox-ADR, %** | 16.1 | 17.6 | 15.6 | 0.543 | 1.157 (0.724-1.849) | 1.10 |
| **SDR, %** | 1.1 | 0.6 | 1.3 | 0.493 | 0.486 (0.059-3.983) | 0.78 |
| **APC, n** | 0.48 | 0.44 | 0.62 | 0.063 |  |  |

**Table S9**- Relationship between hypertension and PDR, ADR, AADR, SDR, and APC.

|  | **All** | **Hypertension** | **No-Hypertension** | **P-value** | **Unadjusted OR (95% CI)**  **(No-Hypertension = ref)** |
| --- | --- | --- | --- | --- | --- |
| **Number, n (%)** | **747** | **321 (43.0)** | **426 (57.0)** |  |  |
| **PDR, %** | 38.7 | 35.8 | 40.8 | 0.163 | 0.809 (0.600-1.090) |
| **ADR, %** | 25.8 | 26.2 | 30.3 | 0.218 | 0.816 (0.590-1.128) |
| **AADR, %** | 12.6 | 12.5 | 12.7 | 0.930 | 0.981 (0.633-1.518) |
| **Prox-ADR, %** | 15.7 | 16.8 | 14.8 | 0.449 | 1.165 (0.784-1.732) |
| **SDR, %** | 0.9 | 0.6 | 1.2 | 0.439 | 0.528 (0.102-2.739) |
| **APC, n** | 0.47 | 0.43 | 0.50 | 0.331 |  |

**Table S10**- Relationship between hyperlipidemia and PDR, ADR, AADR, SDR, and APC.

|  | **All** | **Hyperlipidemia** | **No-Dyslipidemia** | **P-value** | **Unadjusted OR (95% CI)**  **(No-Dyslipidemia = ref)** |
| --- | --- | --- | --- | --- | --- |
| **Number, n (%)** | **703** | **280 (39.8)** | **423 (60.2)** |  |  |
| **PDR, %** | 39.2 | 36.2 | 41.2 | 0.178 | 0.808 (0.593-1.102) |
| **ADR, %** | 29.1 | 27.0 | 30.4 | 0.316 | 0.843 (0.603-1.177) |
| **AADR, %** | 13.1 | 13.5 | 12.9 | 0.818 | 1.053 (0.676-1.642) |
| **Prox-ADR, %** | 15.8 | 15.2 | 16.2 | 0.745 | 0.933 (0.617-1.413) |
| **SDR, %** | 1.1 | 1.1 | 1.2 | 0.895 | 0.908 (0.215-3.828) |
| **APC, n** | 0.49 | 0.49 | 0.50 | 0.925 |  |

**Table S11**- Relationship between CVD and PDR, ADR, AADR, SDR, and APC.

|  | **All** | **No- CVD** | **CVD** | **P-value ^a^** | **Unadjusted OR (95% CI)**  **(No- CVD: ref)** |
| --- | --- | --- | --- | --- | --- |
|  | **763** | **721 (94.5)** | **42 (1.9)** |  |  |
| **PDR, %** | 38.7 | 33.3 | 39.0 | 0.46 | 0.783 (0.405 - 1.513) |
| **ADR, %** | 28.6 | 28.6 | 28.6 | 1.00 | 1.000 (0.502, 1.991) |
| **AADR, %** | 12.3 | 4.8 | 12.8 | 0.12 | 0.342 (0.081 - 1.438) |
| **Prox-ADR, %** | 15.7 | 11.9 | 16.0 | 0.48 | 0.712 (0.274, 1.850) |
| **SDR, %** | 0.9 | 1.0 | 0.0 | 0.52 | 0.998 (0.000, 0.000) |
| **APC, n** | 0.47 | 0.43 | 0.47 | 0.76 | - |

**Table S12**- Relationship between bowel preparation and PDR, ADR, AADR, SDR, and APC.

|  | **All** | **Bad** | **Good** | **P-value ^a^** | **Unadjusted OR (95% CI)**  **(Good: ref)** |
| --- | --- | --- | --- | --- | --- |
| **Number, n (%)** | **1489** | **435 (29.2)** | **1054 (70.8)** |  |  |
| **PDR, %** | 33.4 | 34.0 | 33.2 | 0.761 | 1.037 (0.819-1.314) |
| **ADR, %** | 24.8 | 23.9 | 25.2 | 0.589 | 0.931 (0.717-1.208) |
| **AADR, %** | 9.8 | 8.0 | 10.5 | 0.143 | 0.743 (0.500-1.106) |
| **Prox-ADR, %** | 12.8 | 13.1 | 12.7 | 0.838 | 1.035 (0.743-1.443) |
| **SDR, %** | 0.9 | 1.4 | 0.8 | 0.259 | 1.829 (0.631-5.302) |
| **APC, n** | 0.41 | 0.41 | 0.41 | 0.873 |  |

**Missing data**

**Table S13**- Relationship between center and PDR, ADR, AADR, SDR, and APC; missing data.

|  | **Available data** | **Missing data** | **P-value** |  |
| --- | --- | --- | --- | --- |
| **Number, n (%)** | **2122 (96.1)** | **87 (3.9)** |  |  |
| **PDR, %** | 34.1 | 44.8 | 0.039 | -0.04 |
| **ADR, %** | 25.1 | 28.7 | 0.447 |  |
| **AADR, %** | 10.1 | 9.2 | 0.787 |  |
| **Prox-ADR, %** | 12.9 | 16.1 | 0.388 |  |
| **SDR, %** | 1.0 | 1.1* | 0.883 |  |
| **APC, n** |  |  |  |  |

* 2-sided Fisher’s exact test

**Table S14**- Relationship between colonoscopist and PDR, ADR, AADR, SDR, and APC; missing data.

|  | **Available data** | **Missing data** | **P-value** | **Phi** |
| --- | --- | --- | --- | --- |
| **Number, n (%)** | **2127 (96.3)** | **82 (3.7)** |  |  |
| **PDR, %** | 34.2 | 41.5 | 0.176 | - |
| **ADR, %** | 25.2 | 29.3 | 0.395 | - |
| **AADR, %** | 10.1 | 9.8 | 0.928 | - |
| **Prox-ADR, %** | 12.8 | 18.3 | 0.150 | - |
| **SDR, %** | 1.0 | 0.0* | 0.355 | - |
| **APC, n** |  |  |  |  |

* 2-sided Fisher’s exact test

**Table S15** - Relationship between obesity and PDR, ADR, AADR, SDR, and APC; missing data.

|  | **Available data** | **Missing data** | **P-value** | **Phi** |
| --- | --- | --- | --- | --- |
| **Number, n (%)** | **1514 (68.5)** | **695 (****31.5)** |  |  |
| **PDR, %** | 36.0 | 31.2 | 0.028 | 0.047 |
| **ADR, %** | 26.6 | 22.4 | 0.039 | 0.044 |
| **AADR, %** | 10.9 | 8.2 | 0.050 | 0.042 |
| **Prox-ADR, %** | 14.1 | 10.8 | 0.034 | 0.045 |
| **SDR, %** | 1.1 | 0.7 | 0.491* | - |
| **APC, n** | 0.43 | 0.38 | 0.198 | - |

* 2-sided Fisher’s exact test

**Table S16**- Relationship between diabetes and PDR, ADR, AADR, SDR, and APC; missing data.

|  | **Available data** | **Missing data** | **P-value ^a^** | **Phi** |
| --- | --- | --- | --- | --- |
| **Number, n (%)** | **704 (31.9)** | **1505 (68.1)** |  |  |
| **PDR, %** | 39.3 | 32.2 | 0.001 | 0.07 |
| **ADR, %** | 29.1 | 23.5 | 0.004 | 0.06 |
| **AADR, %** | 12.8 | 8.8 | 0.003 | 0.06 |
| **Prox-ADR, %** | 16.1 | 11.6 | 0.004 | 0.06 |
| **SDR, %** | 1.1 | 0.9 | 0.65* | - |
| **APC, n** | 0.48 | 0.38 | 0.015 | - |

* 2-sided Fisher’s exact test

**Table S17**- Relationship between hypertension and PDR, ADR, AADR, SDR, and APC; missing data.

|  | **Available data** | **Missing data** | **P-value** | **Phi** |
| --- | --- | --- | --- | --- |
| **Number, n (%)** | **747 (33.8)** | **1500 (67.9)** |  |  |
| **PDR, %** | 38.7 | 32.4 | 0.003 | 0.06 |
| **ADR, %** | 25.8 | 23.6 | 0.012 | 0.05 |
| **AADR, %** | 12.6 | 8.8 | 0.005 | 0.06 |
| **Prox-ADR, %** | 15.7 | 11.7 | 0.009 | 0.06 |
| **SDR, %** | 0.9 | 1.0 | 1.0* |  |
| **APC, n** | 0.47 | 0.38 | 0.03 |  |

* 2-sided Fisher’s exact test

**Table S18**- Relationship between hyperlipidemia and PDR, ADR, AADR, SDR, and APC; missing data.

|  | **Available data** | **Missing data** | **P-value** |  |
| --- | --- | --- | --- | --- |
| **Number, n (%)** | **703 (32.1)** | **1506 (67.9)** |  |  |
| **PDR, %** | 39.2 | 32.3 | 0.001 | 0.07 |
| **ADR, %** | 29.1 | 23.5 | 0.05 | 0.06 |
| **AADR, %** | 13.1 | 8.6 | <0.001 | 0.07 |
| **Prox-ADR, %** | 15.8 | 11.7 | 0.008 | 0.06 |
| **SDR, %** | 1.1 | 0.9 | 0.65 |  |
| **APC, n** | 0.49 | 0.38 | 0.007 |  |

**Table S19**- Relationship between CVA and PDR, ADR, AADR, SDR, and APC; missing data.

|  | **Available data** | **Missing data** | **P-value ^a^** | **Phi** |
| --- | --- | --- | --- | --- |
|  | **763 (34.5)** | **1446 (65.5)** |  |  |
| **PDR, %** | 38.7 | 32.3 | 0.003 | 0.06 |
| **ADR, %** | 28.6 | 23.5 | 0.009 | 0.05 |
| **AADR, %** | 12.3 | 8.9 | 0.010 | 0.05 |
| **Prox-ADR, %** | 15.7 | 11.6 | 0.006 | 0.06 |
| **SDR, %** | 0.9 | 1.0 | 0.78 |  |
| **APC, n** | 0.47 | 0.38 | 0.033 |  |

**Table S20**- Relationship between bowel preparation and PDR, ADR, AADR, SDR, and APC; missing data.

|  | **Available data** | **Missing data** | **P-value ^a^** |
| --- | --- | --- | --- |
| **Number, n (%)** | **1489 (67.4)** | **720 (****32.6)** | **-** |
| **PDR, %** | 33.4 | 36.7 | 0.13 |
| **ADR, %** | 24.8 | 26.1 | 0.52 |
| **AADR, %** | 9.8 | 10.6 | 0.58 |
| **Prox-ADR, %** | 12.8 | 13.5 | 0.67 |
| **SDR, %** | 0.9 | 1.1 | 0.70 |
| **APC, n** | 0.41 | 0.43 | 0.65 |
